# Development and psychometric evaluation of the dental care attitudes scale (DCAS)

**DOI:** 10.1186/s12903-025-06655-2

**Published:** 2025-07-23

**Authors:** Márton Lukács, Balázs Fábián, Gábor Papp, Antal Bugán, István Varga

**Affiliations:** 1 Clinical Psychology Center of Clinical Center, University of Debrecen, Debrecen, Hungary; 2 Doctoral School of Dental Sciences, University of Debrecen, Debrecen, Hungary; 3 Department of Behavioural Sciences, Faculty of Medicine, University of Debrecen, Debrecen, Hungary; 4 PractiWork Plc, Budapest, Hungary; 5 Department of Periodontology, Faculty of Dentistry, University of Debrecen, Debrecen, Hungary

**Keywords:** Dental anxiety, Health services, Dental care, Health policy

## Abstract

**Background:**

This study describes the development and assessment of the reliability and validity of the Dental Care Attitudes Scale (DCAS), a new instrument for the measurement of attitudes towards dental care services.

**Methods:**

Scale development employed qualitative and quantitative research methods. The initial set of items was developed from the transcripts of semi-structured interviews conducted with 16 dental patients. The psychometric properties of the DCAS were refined using an initial sample of 132 adults, and then a second sample of 744 participants (non-clinical sample of adults, dental students, and dentists). The five-factor structure was confirmed via CFA in the adult non-clinical sample (*n* = 527). Reliability was assessed by Cronbach’s alpha values. Validity was assessed by examining the correlation coefficients of DCAS scores with established dental health scales, including the Dental Fear Survey, Dental Anxiety Scale, and Dental Belief Survey. Group differences were assessed by Mann-Whitney U and Kruskal-Wallis tests.

**Results:**

The final measure was found to have 17 items, and five factors: Environment, Anesthesia, Delay, Social Status, and System-Related Dissatisfaction. CFA showed good fit (CMIN/DF = 2.11; CFI = 0.94; RMSEA = 0.046), and Cronbach’s alpha ranged from 0.66 to 0.78. The association of the DCAS subscales with other measures were all in line with theoretical expectations, indicating good convergent and divergent validity.

**Conclusion:**

The Dental Care Attitudes Scale is a reliable and valid instrument for assessing attitudes towards dental care. The DCAS identified several attitudes that may hinder dental care attendance and dentist-patient cooperation. By understanding these attitudes, which the DCAS can measure, dentists can address potential issues in clinical practice and improve patient care.

**Supplementary Information:**

The online version contains supplementary material available at 10.1186/s12903-025-06655-2.

## Key points

The Dental Care Attitudes Scale (DCAS) is an important tool that evaluates attitude towards dental care.

The scale showed good validity and reliability.

This tool may aid in research, clinical practice, and health policy decision-making.

**Supplementary Information:**

The online version contains supplementary material available at 10.1186/s12903-025-06655-2.

## Introduction

Oral diseases affect a significant portion of the global population. According to the World Health Organization, approximately 3.5 billion people are impacted, primarily by dental caries, periodontal disease, tooth loss, oral cavity cancer, HIV/AIDS-related oral disease, and oro-dental trauma are major public health problems worldwide. In both developing and developed countries, the burden of oral disease is particularly high among disadvantaged and poor population groups [1–3].

The oral health situation of the Hungarian population is a cause for concern, as evidenced by several epidemiological studies. According to the 2010 Eurobarometer data, an average of 41% of EU citizens have all their natural teeth. However, with only 19%, Hungary ranks last among EU member states [4]. The oral health of the Hungarian elderly population is particularly poor, with high levels of tooth loss, dental caries, and periodontal diseases [5, 6]. The prevalence of impaired oral health-related quality of life in the Hungarian general population is substantial [7]. Additionally, the prevalence of oral diseases, such as tooth decay and periodontal disease, is high among Hungarian adults and elderly population [8, 9].

Despite the increase in dental problems, utilization of dental services is limited. This could be due to various barriers that limit oral health utilization. Dental anxiety and fear associated with irregular or delayed dental care attendance act as barriers to receiving dental care by reducing initial treatment-seeking behavior and causing missed or delayed appointments, even after treatment has been sought [10–12]. Poor communication skills, along with a lack of trust and empathy, can also hinder care-seeking and lead to poor adherence to professional advice, ultimately resulting in lower oral health outcomes [13–16]. However, intrapersonal and interpersonal psychological factors might not be the only causes behind inappropriate dental care utilization. Social-level factors such as financial difficulties, cultural differences, language barriers, and lack of access might also affect dental care attendance [17–20].

Identifying barriers to dental care can provide insights into a broader range of factors that may affect dental care utilization and potentially contribute to policy decision-making and the development of innovative programs aimed at improving access to dental care. Therefore, the purpose of the present study was to explore the attitudes towards the dental care system to identify any attitudes that might affect individuals’ motivation for dental care attendance. To this end, a self-report measure that captures patient attitudes towards dental care was developed and validated.

## Methods

### Design and data collection

The Dental Care Attitudes Scale (DCAS) was developed and validated in two studies: study 1 was qualitative and consisted of semi-structured interviews. Study 2 was quantitative, consisted of two stages and involved the evaluation of the instrument’s psychometric properties.

We utilized our previous qualitative study as a starting point for item generation [21]. Based on semi-structured interviews conducted with 16 dental patients, 128 items were generated and used as the first form of the questionnaire. Each item was carefully worded to reflect the patients’ terminology as much as possible. The questionnaire instructions and a response format were devised. Responses were recorded on a five-point Likert scale ranging from 1 (strongly disagree) to 5 (strongly agree), with higher scores indicating a stronger identification with the corresponding attitude (see Supplementary Material 1). Dental students and dentists were instructed that their responses should reflect the patients’ perspective rather than their own opinions. In the present study, data collection for stages 1 and 2 was based on an online questionnaire compiled by the authors. The survey was administered using Google Forms, an online survey and data-collection software tool. Adult participants from the general population were recruited via a snowball sampling method: initial outreach was conducted through emails and public social media platforms focused on dental health awareness, where individuals were encouraged to complete and share the survey link. Dental students were recruited through convenience sampling by contacting the local University’s Faculty of Dentistry via e-mail. Students were not rewarded for study participation. Reaching dentists was assisted by the Hungarian Dental Association and the Institute for Primary Care and Health Promotion. In addition, there were several collegial requests for participation, and we asked the dentists to share the questionnaire with their colleagues and the survey was also available on social media dentist groups. Inclusion criteria included being an adult (at least 18 years old) and able to read and write in Hungarian.

The study was approved by the Centre’s Regional and Institutional Ethics Committee at the University of Debrecen (registration number: RKEB/IKEB 6044 − 2022, 2022.04.20). The conduct of this study adhered to the principles outlined in the Declaration of Helsinki. Study participants were guaranteed anonymity and assured that the information obtained from them would be kept confidential and would be only used for study purposes. Informed written consent was obtained from all patients before study enrollment.

### Measures

Three questionnaires that are already available in Hungarian as valid and reliable tools and are most closely related to our developed measurement tool based on their measurement focus were selected for validation purposes.

The **Dental Fear Survey** (DFS) was used to measure fear related to dental procedures. It was originally published as a 27-item questionnaire, but it was later condensed to 20 items. In the present study the 20-item version was used. The DFS evaluates the fear induced by dental experiences using a five-point Likert scale, where each question is scored from 1 (low fear) to 5 (very high fear). The total score ranges from 20 to 100 [22]. Scale reliability in the present study was high, with a Cronbach’s alpha coefficient of 0.97.

The **Dental Anxiety Scale** (DAS) is a widely used tool for assessing dental anxiety. It consists of four questions, each scored on a scale from 1 (low anxiety) to 5 (high anxiety), resulting in a total score ranging from 4 to 20. Scores of 13 or above indicate significant dental anxiety, while scores between 9 and 12 suggest moderate anxiety, and scores below 9 reflect low anxiety. The DAS has proven to be a reliable and valid measure of dental anxiety, with high internal consistency and test-retest reliability [23, 24]. In the present study, scale reliability was very good (Cronbach’s alpha = 0.88).

The **Dental Belief Survey** (DBS) is designed to evaluate patients’ attitudes towards dental professionals and dental treatment. This survey consists of 15 items divided into three subscales: Professionalism, Communication, and Lack of Control. Responses are recorded on a five-point Likert scale ranging from 1 (not negative at all) to 5 (highly negative), with higher scores indicating more negative perceptions. Since previous studies found different factor structures in different samples, in the present study only the total score was calculated [25, 26]. Internal consistency (Cronbach’s alpha) was 0.88 for the DBS.

### Statistical analysis

Statistical analyses were performed using R version 4.4.0 software and SPSS v. 22 (SPSS Inc., IBM, Chicago, IL, USA) software. The normality of the data was checked by the Kolmogorov-Smirnov test. The rate of missing data was below 0.001%. Missing data were replaced by the respective sample mean for continuous variables. Associations with validity measures were assessed using Pearson’s correlation coefficients or Spearman’s rank order correlation coefficients, as appropriate. The Mann-Whitney U test was used to make comparisons between two groups. Kruskal-Wallis test was used to examine the differences across multiple groups.

Factor analysis (principal component extraction and promax rotation) of the initial items was used to identify the most appropriate items. Factor generation was exploratory (EFA), so no restriction was placed on the number of factors to be extracted. Internal reliability was assessed using Cronbach’s alpha coefficients. Items were systematically removed one at a time, in accordance with the following criteria: items with high loadings on more than one factor (factor loadings over 0.3 on at least two scales) and items demonstrating factor loading below 0.4 were eliminated. After removing items from the item pool, EFA was repeated until every item met the criteria presented above. Additionally, each identified domain was examined to see if removal of any item led to an increase in its internal consistency.

After removing poorly performing items, confirmatory factory analysis (CFA) was used to assess the optimal simple structure of the final form of the scale. While EFA was conducted on the full dataset, the CFA was conducted only in the adult non-clinical sample (*n* = 527) of the second stage of study 2.

After removing poorly performing items, confirmatory factor analysis (CFA) was conducted on the adult non-clinical subsample (*n* = 527) from the second stage of Study 2 to evaluate the optimal simple structure of the final scale, following EFA on the full dataset. Criteria for good model fit were judged by using the Relative chi-square index (CMIN/DF), the goodness of fit index (GFI), the Tucker-Lewis index (TLI), Comparative fit index (CFI), the root mean square error of approximation (RMSEA), and the Standardized root means residual (SRMR). Following the recommendations of several authors [27–30], the following criteria were used to indicate the fit of the model to the data: CMIN/DF below 3.00, GFI, CFI, TLI > 0.90; SRMR and RMSEA < 0.08. Reset of the data analysis was conducted in the three samples of the second stage of the study.

The convergent and divergent validity of the DCAS were assessed by examining the association of DCAS scores with well-established dental health related scales available in Hungarian in the three samples (adult non-clinical sample, dental student, dentist) in the second stage of the study. Our expectation was that the DCAS subscales will show stronger correlations with theoretically related constructs, weaker correlations with theoretically less-related constructs, and negative correlations with constructs with inverse measurement functions. Specifically, we expected the Environmental Anxiety subscale demonstrating strong significant correlations (from 0.5 to 0.9) with DFS and DAS in the correlation analysis. We predicted that the Delay subscale will show significant moderate or string correlations (from 0.3 to 0.7) with the Environment subscale and with DFS and DAS scores. DBS was expected to have lower but still significant and moderate (from 0.3 to 0.5) association with the Environment and Delay subscales than DFS and DAS. Anesthesia, Social Status, System related dissatisfaction subscales were expected to show low significant correlations (0.1 to 0.3) with DFS, DAS and DBS.

## Results

In the first stage of the study, in the adult non-clinical sample 67.42% of the participants were women. The mean age was 35.8. In the second stage of the study, there was a total of 744 respondents, which consisted of three subgroups: adult non-clinical sample (*n* = 527), dental student sample (*n* = 114) and dentists sample (*n* = 103) (see Table 1).

**Table 1:** Socio-demographic characteristics

	First stage	Second stage			
	Adult non-clinical sample (*n* = 132)	Adult non-clinical sample (*n* = 527)	Dental student (*n* = 114)	Dentist (*n* = 103)	Total (*N* = 744)
Age, average ± Standard deviation	35.8 ± 11.92	32.79 ± 12.15	22.13 ± 2.78	41.51 ± 12.7	32.36 ± 12.47
Gender					
Female	89 (67.42%)	378 (71.73%)	83 (72.81%)	67 (65.05%)	528 (71%)
Male	43 (32.58%)	149 (28.27%)	31 (27.19%)	36 (35.95%)	216 (29%)
Highest educational attainment					
Primary school	1 (0.76%)	1 (0.19%)	0	0	1 (0.1%)
Secondary school	36 (27.27%)	185 (35.1%)	114 (100%)	0	299 (40.19%)
University	95 (71.97%)	341 (64.71%)	0	103 (100%)	444 (59.7%)
Residence					
Village	NA	57 (10.82%)	18 (15.79%)	8 (7.77%)	83 (11.2%)
City	NA	171 (32.45%)	59 (51.75%)	41 (39.81%)	271 (36.4%)
State capital	NA	205 (38.9%)	34 (29.83%)	35 (33.98%)	274 (36.8%)
Country capital	NA	94 (17.84%)	3 (2.63%)	19 (18.45%)	116 (15.6%)

In the first stage, the original 128 generated items were reduced to 70 items, based on factor loadings and internal reliability indexes. Following the same steps, after the second data collection 5 factors and 17 items were identified as an appropriate structure for DCAS. For the final confirmatory factor analysis in the adult non-clinical sample (*n* = 527), the Kaiser-Meyer-Olkin (KMO) value was 0.713, exceeding the recommended value of 0.6. Bartlett’s Test of Sphericity was statistically significant (χ2 = 2145.82; *p* <.001), supporting the factorability of the final correlation matrix. The five-factor structure accounted for 61.16% of the overall variance within the data set. Findings clearly indicated good fit for the five-factor model: CMIN/DF = 2.11, GFI = 0.95, TLI = 0.93, CFI = 0.94, RMSEA = 0.046, SRMR = 0.045. The domains identified were Environmental Anxiety (4 items), Anesthesia (4 items), Procrastination (3 items), Social Status (3 items), and System related dissatisfaction (3 items). The Environmental Anxiety domain measures the anxiety caused by the dentist’s office and the sight, the sound, and the touch of medical equipment. The Anesthesia subscale consists of items reflecting one’s fear of losing control due to anesthesia. The Delay subscale measures the extent to which dental care is delayed due to financial constraints. The Social Status subscale measures the belief that missing teeth indicates lower socioeconomic status. System related dissatisfaction measures one’s dissatisfaction with the low financial coverage of the social security based state dental care system. The internal consistency for the five subscales were alfa = 0.71, 0.78, 0.75, 0.66 and 0.67, respectively. The results of reliability analysis, factors loadings and descriptive statistics are shown in Table 2.

**Table 2:** Results from reliability and factor analysis in the adult non-clinical sample (*n* = 527)

Items in English (items in Hungarian)	Reliability			Rotated loadings from ten-factor solution					Mean (Standard deviation)
	CITC	Alpha if deleted	ENV		AN	DEL	STA	SYS	
7. I feel sick from the smell of the dentist’s office. (7. Rosszul vagyok a fogorvosi rendelő szagától.)	0.55	0.62	0.68						2.40 (1.46)
1. I cannot relax in the dentist’s chair. (1. A fogorvosi székben nem tudok ellazulni.)	0.55	0.62	0.68						3.01 (1.48)
13. The scariest thing for me is the sound of the dental drill. (13. A legfélelmetesebb számomra a fogorvosi fúró hangja.)	0.47	0.67	0.59						2.87 (1.43)
12. I don’t like seeing the doctor draw up the anesthetic. (12. Nem szeretem látni, amikor az orvos felszívja az érzéstelenítőt.)	0.44	0.69	0.54						2.94 (1.54)
11. I don’t want to be put under anesthesia because I want to know what’s happening to me. (11. Ne altassanak, mert tudni akarom, mi történik velem.)	0.64	0.69			0.75				3.24 (1.40)
6. No matter how afraid I am of dental procedures, I would never agree to anesthesia. (6. Bármennyire is félek a fogászati beavatkozástól, az altatást soha nem vállalnám.)	0.63	0.70			0.75				2.75 (1.37)
4. Anesthesia causes much more harm than enduring a little pain. (4. Az altatás sokkal több kárt okoz, mint az, hogy egy kis fájdalmat el kell viselnem.)	0.54	0.75			0.63				3.17 (1.28)
14. Anesthesia doesn’t allow me to control what’s happening to me, so I’m afraid of it. (14. Az altatás nem teszi lehetővé, hogy kontrolláljam, mi történik velem, ezért félek tőle.)	0.53	0.75			0.62				2.75 (1.38)
9. I can’t afford modern dental treatments because of my financial situation. (9. A modern fogászati kezeléseket anyagi helyzetem miatt nem érhetem el.)	0.64	0.59				0.82			2.78 (1.44)
8. My teeth are neglected because I can’t afford dental treatment. (8. Azért elhanyagoltak a fogaim, mert a fogászati kezelést nem tudom megfizetni.)	0.57	0.68				0.72			2.05 (1.30)
10. I’m not satisfied with my teeth; if I had the money, I would go for cosmetic procedures. (10. Nem vagyok elégedett a fogaimmal, ha lenne pénzem, belevágnék az esztétikai célú beavatkozásokba.)	0.54	0.72				0.58			2.81 (1.52)
17. Missing teeth symbolize social disadvantage. (17. A hiányos fogsor a társadalmi elesettség jelképe.)	0.56	0.45					0.78		3.19 (1.23)
15. Missing teeth are a sign of poverty. (15. A hiányos fogsor a szegénység jele.)	0.53	0.48					0.74		2.80 (1.28)
5. Missing teeth suggest negligence. (5. A hiányos fogsor igénytelenségre utal.)	0.34	0.74					0.43		3.22 (1.27)
2. There should be social insurance support for modern dental prosthetic procedures. (2. Szükség lenne a modern fogpótló eljárások TB támogatására.)	0.57	0.48						0.83	4.52 (0.85)
3. Modern prosthetic dentistry procedures are unjustifiably overpriced. (3. A modern fogpótlástani eljárások indokolatlanul túlárazottak.)	0.47	0.58						0.58	4.13 (0.98)
16. I believe social insurance should also support cosmetic dental procedures to some extent. (16. Szerintem a társadalombiztosításnak az esztétikai jellegű fogászati beavatkozásokat is támogatnia kellene valamilyen mértékben.)	0.42	0.67						0.51	4.17 (1.04)

Note. Factor loadings under 0.3 are not shown. ENV = Environmental Anxiety, AN = Anesthesia, DEL = Delay, STA = Social Status, SYS = System related dissatisfaction, CITC = corrected item-total correlation

Correlation analysis (Spearman’s rho) showed that the age weakly, but significantly correlated with DEL (ρ = − 0.10, *p* <.01), STA (ρ = 0.10, *p* <.01), and SYS (ρ = 0.15, *p* <.01) subscales. An independent samples Mann–Whitney U-test showed that the difference between the ENV (*p* =.005), DEL (*p* =.004) and SYS (*p* <.001) scores of females (M = 12.71, SD = 4.28; M = 8.79, SD = 3.51; M = 12.78, SD = 2.23) and males (M = 11.70, SD = 4.40; M = 7.99, SD = 3.30; M = 11.91, SD = 2.59) was statistically significant, respectively.

An independent samples Kruskal–Wallis test showed a significant difference in DEL scores regarding current residence (*p* =.033). The difference was further investigated in accordance with the hypotheses by post hoc comparisons using Mann–Whitney U-tests. Post hoc Mann–Whitney U-tests showed that the DEL scores of State capital residents (M = 8.17, SD = 3.71) were significantly different (*p* =.04; *p* =.01) than for village (M = 9.11, SD = 2.87) and city (M = 8.89, SD = 3.33) residents, respectively.

Mann–Whitney U-tests showed that the DEL scores of study participant with university level education (M = 9.46, SD = 3.4) were significantly lower (*p* <.001) than participant with middle school level education (M = 7.96, SD = 3.39). Since only one person had only primary education, that subgroup was excluded from analysis.

**Table 3:** Spearman r correlations between the DCAS subscales and the DFS, DAS, and DBS measures

	ENV	AN	DEL	STA	SYS
ENV	1.00				
AN	− 0.08*	1.00			
DEL	0.31**	− 0.06	1.00		
STA	0.15**	0.09*	0.16**	1.00	
SYS	0.11**	0.02	0.14**	0.12**	1.00
DFS	0.74**	− 0.12**	0.37**	0.10**	0.10**
DAS	0.69**	− 0.11**	0.31**	0.10**	0.14**
DBS	0.39**	− 0.11**	0.37**	0.09*	0.03

ENV = Environmental Anxiety, AN = Anesthesia, DEL = Delay, STA = Social Status, SYS = System related dissatisfaction, DFS = Dental Fear Survey, DAS = Dental Anxiety Scale, DBS = Dental Beliefs Survey, ENV = Environmental Anxiety, AN = Anesthesia, DEL = Delay, STA = Social Status, SYS = System related dissatisfaction, **: *p* <.01, *: < 0.05

Correlation analysis was done to analyze the connection between DCAS subscales and DFS, DAS and DBS. The correlations presented in Table 3 are all in line with the theoretical expectations indicating good convergent and divergent validity of DCAS. The correlations between DCAS domains were weak and did not always show significant correlations. The ENV subscale showed the strongest significant (*p* <.01) correlations with the DFS, DAS and DBS subscales (ρ = 0.80; ρ = 0.74; ρ = 0.45), respectively. DEL showed moderate significant (*p* <.01) correlations with the DFS, DAS and DBS subscales (ρ = 0.37; ρ = 0.31; ρ = 0.37), respectively. AN, STA and SYS subscales showed weak significant correlations with the other scales (from ρ = 0.10 to ρ = 0.14) and in some cases the association did reach the level of significance.

**Table 4:** DCAS subscale mean scores and standard deviations in the three study groups

	Adult *N* = 527	Student *N* = 114	Dentist *N* = 103	F	*p*
ENV	11.22 (4.33)^b, c^	14.93 (2.34)^a^	15.75 (2.90)^a^	141.65	< 0.001
AN	11.90 (4.21)^c^	11.14 (3.31)	10.45 (3.57)^a^	13.55	= 0.001
DEL	7.64 (3.49)^b, c^	11.09 (2.11)^a^	10.47 (2.20)^a^	133.11	< 0.001
STA	9.22 (2.92)^b, c^	9.99 (2.70)^a^	10.78 (2.77)^a^	26.24	< 0.001
SYS	12.83 (2.23)^b^	11.28 (2.49)^a, c^	12.37 (2.50)^b^	44.45	< 0.001

ENV = Environmental Anxiety, AN = Anesthesia, DEL = Delay, STA = Social Status, SYS = System related dissatisfaction

Superscripts (a, b, c) denote pairs of groups differences, a = Healthy adults; b = Student group; c = Dentist group

Mean DCAS subscale scores in the three study subgroups were compared to assess differences (see Table 4). A series of Kruskal-Wallis tests with Mann-Whitney pairwise comparisons showed significant differences between subgroups in every DCAS domain. Respectively, the adult group had significantly lower ENV (*p* <.001), PRO (*p* <.001) and STA (*p* <.001; *p* =.027) scores than the adult and the dentist groups, while there were no differences between students and dentists. Adult and student groups showed no significant differences in AN scores, while there was a significant difference (*p* =.002) between the dentists and the adult group members. Regarding SYS scores, there was no difference between adult and dentist groups, while student group scores were significantly lower (*p* <.001; *p* <.002, respectively).

## Discussion

The primary purpose of our study was to develop and validate a psychometrically sound measure of dental care attitudes. Based on the results of the confirmatory factor analysis conducted in the adult non-clinical sample (*n* = 527) and the Cronbach’s alpha values, we conclude that DCAS demonstrates good reliability, validity, and generalizability. Through the item selection process, five components of attitudes were identified: Environmental Anxiety, Anesthesia, Delay, Social Status and System related dissatisfaction. After the factor structure was confirmed in the adult non-clinical sample, differences in dental care attitudes were examined descriptively through group comparisons - among adults, dental students, and dentists - providing preliminary support for the measure’s generalizability across these groups. While dental anxiety [31] and avoidance behavior [32] have been extensively studied and received significant attention before, the role of the other social level factors has not been widely assessed. To the best of our knowledge, the only relevant attitude scale, The Dental Attitudes Questionnaire (DAQ), was developed and finalized in 1987 in the Netherlands [33]. The healthcare attitudes it identified were mostly different from the motives found in our study: cynicism, health concern, motivation, oral function, social aesthetics, susceptibility, halo effect, and infrequency. The study reporting the development of DAQ was conducted over 40 years ago in a different population, country and cultural background, where dental practices and the healthcare system were likely, and probably substantially, different. These differences may have influenced the motivational background of dental care attitudes. Translating the DCAS and DAQ to other languages and conducting cross-cultural comparison studies might be a fruitful direction for further research.

Sociodemographic characteristics such as age, gender, residence and education level showed significant association with some DCAS subscales; however, the strength of these connections was less than moderate. We argue that these characteristics do not seem to be very important in determining dental care attitudes, although in some circumstances they might show a stronger influence on them. We would only highlight that delaying dental care attendance (Delay subscale) showed a significant association with most of the sociodemographic variables. In previous studies avoidance was related to age, gender, residence and education level [34–38]. Therefore, we would recommend collecting related data when studying procrastination and avoidance behavior.

We hypothesized that the DCAS subscales would exhibit stronger correlations with constructs that are theoretically related, weaker correlations with those that are less related, and negative correlations with constructs measuring opposing functions. The results supported these preliminary assumptions indicating acceptable convergent and divergent validity. The Environment subscale showed a moderate association with DFS (ρ = 0.45, *p* <.01) and strong correlations with DFS and DAS (ρ = 0.80, *p* <.01; ρ = 0.74, *p* <.01, respectively). In the correlation analysis the Environment subscale showed a very strong association with the measures of dental fear and anxiety, since it measures the experience of the anxiety provoking nature of the dentist’s office. The Delay subscale showed a significant association with the Environment subscale (ρ = 0.34, *p* <.01). The Delay subscale also showed moderate significant correlations with the DFS and DAS scores (ρ = 0.37, *p* <.01; ρ = 0.31, *p* <.01, respectively). Even though participants stated that they postpone dental care attendance due to financial reasons, based on the results we would argue that they rationalize their avoidance behavior by pointing out financial constraints; however, they do experience some anxiety at some level without admitting it. These findings align with previous research demonstrating that dental fear and anxiety can lead to dental care avoidance [10]. The other three DCAS subscales - Anesthesia, Social Status, System related dissatisfaction - represent theoretically distinct groups of attitudes, and they have only a marginal association with the other scales. The very low correlations observed between theoretically unrelated constructs - e.g. the association of STA (attitudes toward the social status of good/bad oral health) and DAS (ρ = 0.10, *p* <.01) - also provided support for scale validity. Strong significant negative correlations were not observed between the variables. These results are in line with our prior expectations confirming the psychometrically sound nature of DCAS. Based on the attitudes identified in the present study we hypothesize that the financial possibilities of the general population, the range of free dental procedures and the costs of those dental procedures that are not supported by social insurance are not balanced. It might be appropriate to suggest policy changes aiming at broadening the range of free dental procedures and/or keeping the costs of more special and advance dental procedures within the financial limits of a larger proportions of the country’s population.

According to dentists and dental students, healthy adults are more afraid of the anxiety-inducing effects of seeing, hearing, and physically experiencing dental instruments, as well as the overall context of the dental office, than patients themselves actually are. Therefore, dental students and dentists might overestimate the actual anxiety experienced by patients and pay less attention to other aspects of the doctor-patient interaction. There is no significant difference between dentists and students in this regard. Healthy adults want to maintain and have more control than dentists think they do. It seems that patients have a need to control the circumstances and progress of their treatment. Dentists may potentially underestimate how necessary it is to establish a partnership with the patient. Although many previous studies have shown that autonomy, partnership and involving the patient in the decision making are important for patients [39–41], it is worth emphasizing to practicing dentists that, even though patients do not need experience physical control, they do need it on a psychological level. Both dentists and students equally believe that adults are much more likely to procrastinate in seeking dental care than healthy adults themselves think they are. We can assume that during their practice, students, and during their work, dentists - who mostly work in public healthcare, potentially with patients of lower socioeconomic status - often encounter patients who do not attend regular check-ups and procrastinate a lot (at least partially due to financial reasons). Among the adults in our sample, there may be more individuals in a better financial situation, leading to less procrastination, and it is also possible that they are more likely to seek private care. Regarding the Social Status subscale, which measures the social representation of missing teeth, we can see that adults are more tolerant of individuals with missing teeth than students and dentists think they are. Dental students underestimated, while dentists accurately assessed, what healthy adults think about the lack of funding for specialized dental care. In university education, this is probably not a core part of the curriculum, and thus students could not accurately assess the importance of this issue. Furthermore, the students are younger and have probably had fewer occasions to pay for dental care out of their own pocket since their parents likely paid for it, so they may not fully understand the financial background.

Several differences between the three study groups were identified indicating that the dental students’ and dentists’ perceptions do not necessarily reflect actual patient experiences and attitudes. These results are well in line with previous studies demonstrating that it is difficult for dental professionals to estimate patient anxiety without using measurement tools [42] and that dentists and patients differ in their perception of anxious dental visual stimuli [43]. These findings support the validity of our results and the psychometric strength of DCAS. These results highlight the need for improved communication and perspective-taking in dental practice. Integrating these findings into dental education could help students develop better strategies for understanding and managing patient experiences. We recommend incorporating interview techniques and validated measures, such as the DCAS, into dental training programs to enhance awareness of patient perspectives. In the long term, the integration of such methods could improve patient-centered care and reduce the risk of misinterpreting patient concerns.

Although this study provides important evidence regarding patients’ attitudes towards dental care, certain limitations must be acknowledged. There is a possibility of selection bias due to using an online survey format and convenience sampling procedure with the lack of randomization. Hence, the generalization of the results should be considered with caution and further research may consider using a randomized sampling technique. Secondly, only attitudes were measured on a cognitive level, behavior level assessment was not applied. We would recommend further studies to investigate the relationship between dental care attitudes and socioeconomic status, dental care attendance (e.g. regular screening participation), avoidance behavior, oral health, and patient satisfaction. Additionally, since the study samples were not matched, data were not collected from patients who were specifically treated by the dentists involved in the study. Therefore, the observed differences between the three groups should be interpreted accordingly. Our goal was to capture professionals’ generalized perceptions of patient attitudes rather than a specific patient they have interacted with. It is recommended that future studies investigating differences between specific dentists’ and patients’ attitudes to use matched samples of dentists and their actual patients to allow for methodologically sound comparisons.

## Conclusion

To conclude, a questionnaire was developed in this study to allow detailed assessment of attitudes toward dental care. It was found that DCAS demonstrated reliability and validity in three samples: adults, dental students, and dentists. The present study revealed several attitudes that might form barriers to dental care attendance and dentist-patient cooperation. Using DCAS in daily clinical practice may help dentists to adequately address these potential issues. Our findings provide a reasonable justification for the use of this measure in future research and have implications for interventions designed to change attitudes and policy development aiming at increasing dental care utilization and improving dental care quality and general oral health.

## Electronic supplementary material

Below is the link to the electronic supplementary material.

Supplementary Material 1

## Data Availability

Research data available from the first author upon reasonable request.
