# Supplementary material for: Development and psychometric evaluation of the dental care attitudes scale (DCAS)
Source: BMC Oral Health. 2025 Jul 23;25:1235. doi: 10.1186/s12903-025-06655-2 (PMC12288335; doi:10.1186/s12903-025-06655-2)
Supplement: Supplementary file 1 — Supplementary Material 1 [file 12903_2025_6655_MOESM1_ESM.pdf]

Dental Care Attitudes Scale (DCAS)

Instructions:

In the following, you can rate situations related to dental treatment on a five-point scale, where you can express your level of agreement or disagreement.

Instructions for dentists:

In the following, you can assess your clients' attitudes toward various dental situations.

	Strongly disagree 1	Partially disagree 2	I can't decide 3	Partially agree 4	Strongly agree 5
1. I cannot relax in the dentist's chair.					
2. There should be social insurance support for modern dental prosthetic procedures.					
3. Modern prosthetic dentistry procedures are unjustifiably overpriced.					
4. Anesthesia causes much more harm than enduring a little pain.					
5. Missing teeth suggest negligence.					
6. No matter how afraid I am of dental procedures, I would never agree to anesthesia.					
7. I feel sick from the smell of the dentist's office.					
8. My teeth are neglected because I can't afford dental treatment.					
9. I can't afford modern dental treatments because of my financial situation.					
10. I'm not satisfied with my teeth; if I had the money, I would go for cosmetic procedures.					
11. I don't want to be put under anesthesia because I want to know what's happening to me.					
12. I don't like seeing the doctor draw up the anesthetic.					
13. The scariest thing for me is the sound of the dental drill.					
14. Anesthesia doesn't allow me to control what's happening to me, so I'm afraid of it.					
15. Missing teeth are a sign of poverty.					
16. I believe social insurance should also support cosmetic dental procedures to some extent.					
17. Missing teeth symbolize social disadvantage.					
